# Exploring the independent factors influencing prolonged QTc intervals in patients with acute-on-chronic liver failure caused by hepatitis B

**DOI:** 10.1097/MD.0000000000050941

**Published:** 2026-10-02

**Authors:** Rou Zhang, Heng Gao, Mengjun Fu, Juan Li, MiMi Zhou, YaLi Feng, JinFeng Liu, YingRen Zhao, Yingli He

**Affiliations:** a Department of Infectious Diseases, The First Affiliated Hospital of Xi’an Jiaotong University,Xi’an, Shaanxi, China; b Department of Xi’an Health School, Xi’an, Shaanxi, China.

**Keywords:** Acute-on-chronic liver failure, Disease severity, Hepatitis B virus, QTc interval, Risk factors

## Abstract

Prolonged QTc interval is a common electrophysiological abnormality in patients with chronic liver disease, but limited evidence exists regarding its occurrence in acute-on-chronic liver failure (ACLF), particularly in hepatitis B virus–related ACLF (HBV-ACLF). Given the rapid progression, high mortality, and severe complications associated with ACLF, understanding cardiac electrophysiological changes is clinically important. This study aimed to determine the incidence of QTc prolongation in patients with HBV-ACLF and to identify independent factors associated with QTc abnormalities. A retrospective study was conducted on 206 consecutive patients with HBV-ACLF admitted between January 2016 and December 2023. Heart rate and QT interval data were obtained from 12-lead electrocardiograms performed at admission. QTc was calculated using Bazett’s formula. Patients were categorized into prolonged and non-prolonged QTc groups using a cutoff of 440 ms. Demographic, clinical, and laboratory variables were compared, and significant factors (*P* < .05) were entered into multivariate logistic regression to determine independent predictors. Of the 206 patients included, 68 (33%) exhibited prolonged QTc intervals. Univariate analysis revealed significant differences in red blood cell count (RBC), total cholesterol (CHOL), creatinine (CRE), potassium (K), fibrinogen (FIB), ascites, HBV-DNA level, and Child–Pugh classification (all *P* < .05). Multivariate logistic regression identified K, CRE, HBV-DNA, and ascites as independent predictors of QTc prolongation (*P* < .05 for all). Approximately one-third of patients with HBV-ACLF demonstrated prolonged QTc intervals. Electrolyte imbalance, renal dysfunction, viral replication activity, and ascites were independently associated with QTc prolongation. Routine QTc monitoring may provide a simple and noninvasive tool for early identification of patients at increased risk of cardiac complications.

## 1. Introduction

Liver failure, primarily induced by viruses, alcohol, drugs, or ischemia–reperfusion injury, represents the most severe manifestation of hepatic damage. It results in significant impairment of essential liver functions, including detoxification, biotransformation, synthesis, and excretion, and is accompanied by multiple severe complications such as ascites, hepatic encephalopathy, coagulation disorders, jaundice, hepatorenal syndrome, and cardiac arrhythmias.^[1,2]^Based on disease progression and histopathological characteristics, liver failure is currently classified into 4 major types: acute, subacute, slow-onset acute, and chronic liver failure.

In Western countries, drugs are the predominant cause of acute and subacute liver failure, whereas chronic liver failure is primarily related to alcohol consumption. In China, hepatitis viruses – especially hepatitis B virus (HBV) – remain the leading causes, accounting for more than 80% of cases. Other important factors include drug- and alcohol-induced injury. Acute-on-chronic liver failure (ACLF) constitutes the majority of liver failure cases.^[3,4]^

With advances in medical technology and the widespread implementation of hepatitis B vaccination, both the incidence and mortality of liver diseases have declined. However, patients with slow-onset acute liver failure continue to present a major global clinical challenge, characterized by rapid onset, multiple complications, high cost, high mortality, and limited treatment options.

Cardiovascular complications have emerged as a critical contributor to increased mortality in liver failure. They represent the third leading cause of death in these patients, following transplant rejection and infection. Among them, QTc prolongation is the most frequent electrocardiographic abnormality. Epidemiological studies report that up to 50% of patients with liver disease exhibit cardiac abnormalities.^[5]^These patients are particularly prone to malignant arrhythmias and sudden cardiac death, which are closely associated with higher mortality.^[6,7]^QTc prolongation is recognized both as a risk factor for ventricular arrhythmias and as an electrophysiological hallmark of cirrhotic cardiomyopathy.^[8,9]^

Most prior studies have focused on QTc intervals in cirrhotic patients. In Western populations, cirrhosis is often associated with alcohol or drug use, whereas in China, HBV-related cirrhosis predominates. Therefore, some existing conclusions may not be directly generalizable to Chinese patients. Moreover, subacute and chronic liver failure are the most common clinical patterns among Chinese adults, both exhibiting rapid onset, multiple complications, high mortality, and poor prognosis.

Consequently, this study aimed to investigate the prevalence of QTc prolongation and its independent influencing factors in Chinese patients with HBV-related ACLF.

## 2. Material and methods

### 2.1. Object of study

This retrospective case–control study was conducted at a single center and included data from 206 patients diagnosed with HBV-related acute-on-chronic liver failure (HBV-ACLF) who were treated in the Department of Infection, First Affiliated Hospital of Xi’an Jiaotong University, between January 2016 and December 2023.

All patients met the diagnostic criteria for ACLF outlined in the Guidelines for the Diagnosis and Treatment of Liver Failure (2018 Edition), which defines ACLF as a syndrome characterized by acute jaundice and coagulopathy triggered by various insults in the setting of chronic liver disease. Complications may include hepatic encephalopathy, ascites, electrolyte imbalance, infection, hepatorenal syndrome, hepatopulmonary syndrome, and multi-organ failure.

The inclusion criteria were as follows: rapid progression of jaundice; total bilirubin ≥ 10 × ULN or a daily increase ≥ 17.1 μmol/L; and bleeding manifestations with prothrombin time activity (PTA) ≤ 40% (or INR ≥ 1.5).

Exclusion criteria included: ACLF not caused by HBV infection; presence of cardiovascular diseases such as arrhythmia or heart failure; other significant organ dysfunctions; use of medications known to affect QTc intervals during hospitalization; and absence of ECG data during admission.

After screening, 206 patients were enrolled: 68 in the prolonged QTc group and 138 in the non-prolonged group. The flow chart is shown in Figure 1.

**Figure 1.**
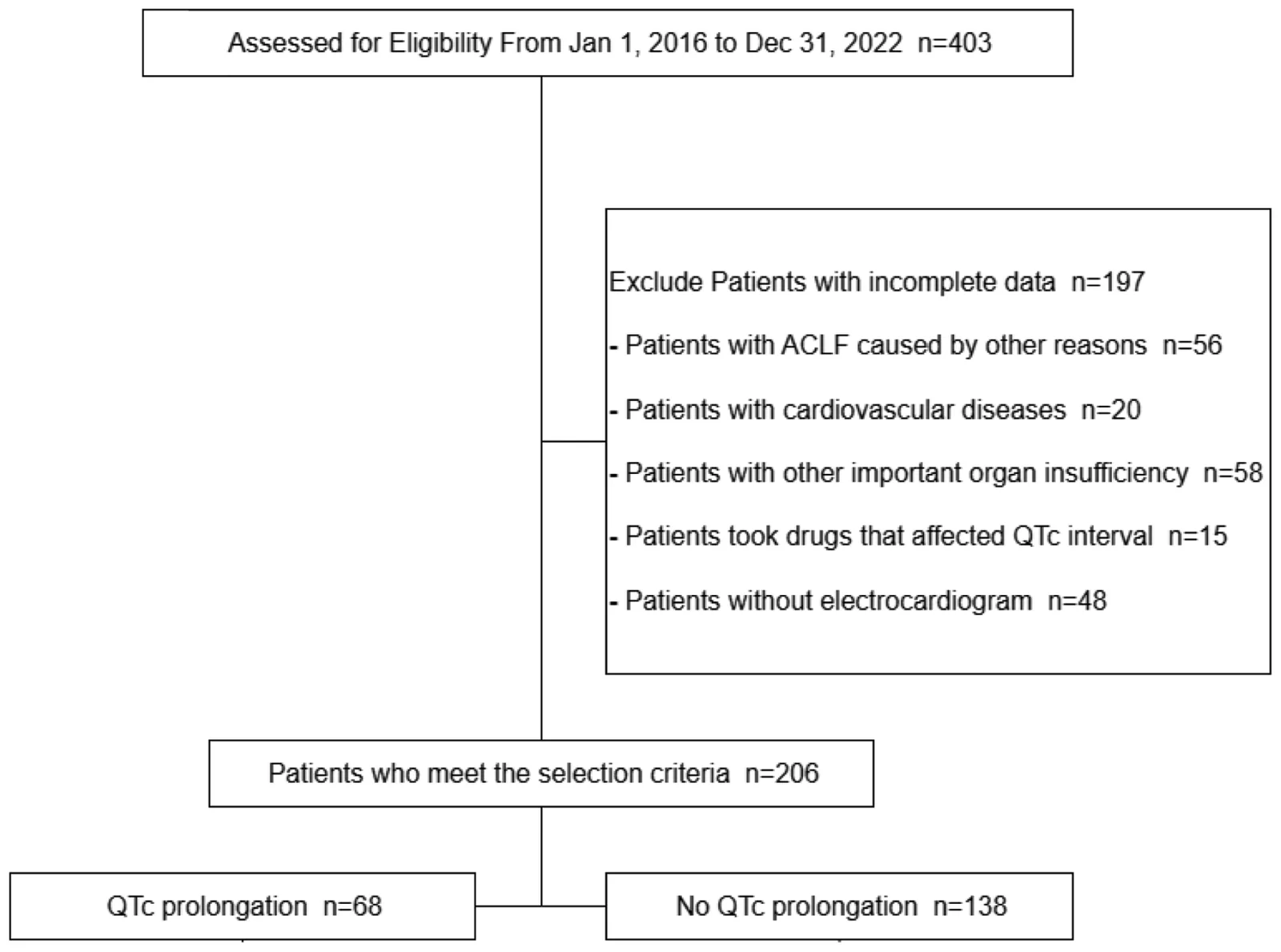
Flow chart of patient selection and inclusion for the HBV-ACLF cohort.

The study protocol was approved by the Ethics Committee of Xi’an Jiaotong University (approval number: XJTU1AF2018LSL-002) and conducted in accordance with the principles of the Declaration of Helsinki. Written informed consent was obtained from all participants.

### 2.2. Research method

Data from 12-lead electrocardiograms (ECG) obtained after admission for each patient were meticulously gathered. The corrected QTc interval was calculated using the widely accepted Bazett formula (QTc = QT/RR; RR = 60/HR(heart rate)).^[10]^Based on the corrected QTc, patients were categorized into 2 groups: prolonged QTc (> 440 ms) and non-prolonged QTc (≤ 440 ms).^[11]^

Comprehensive clinical data were collected for each patient, including age, sex, presence of ascites or hepatic encephalopathy, HBV-DNA level (quantified by high-sensitivity assays; threshold: 20 IU/mL), and laboratory parameters: red blood cell count (RBC), white blood cell count (WBC), hemoglobin (HGB), aspartate aminotransferase (AST), alanine aminotransferase (ALT), total cholesterol (CHOI), albumin (ALB), creatinine (CRE), potassium (K), fibrinogen (FIB), prothrombin time activity (PTA), Child–Pugh classification (CTP), and Model for End-Stage Liver Disease (MELD) score.

### 2.3. Statistical analysis

Statistical analysis of all variables was performed using IBM SPSS Statistics version 26.0. The normality of the metric data was assessed using the Shapiro–Wilk test. For normally distributed metric data, an independent sample t-test was used to conduct statistical analysis between the case and control groups, presented as mean ± standard deviation. Non-normally distributed data were analyzed using nonparametric tests and are presented as median (interquartile range).

Categorical variables were analyzed using the χ^2^ test or Fisher’s exact test. Variables with *P* < .05 in univariate analysis were entered into binary logistic regression to identify independent predictors of QTc prolongation. Statistical significance was set at *P* < .05.

## 3. Results

A total of 403 patients were initially diagnosed with acute-on-chronic liver failure during the study period. Of these, 197 were excluded for not meeting the inclusion criteria. Ultimately, 206 patients were enrolled in the final analysis, among whom 68 (33%) exhibited prolonged QTc intervals. Baseline characteristics of the included patients are summarized in Table 1.

**Table 1:** Baseline characteristics of patients with HBV-ACLF.

Variables	All patients		
	Number of patients(n)	Mean ± SD or frequency(percentage)	Median(Range)
Sex(male/female)	206	177/29	
HBV-DNA(positive/negative)	206	189/17	
Ascites(positive/negative)	206	110/96	
Hepatic encephalopathy(positive/negative)	206	35/171	
Age(years)	206	42.58 ± 11.07	42 (34~50.25)
RBC(10^12^/L)	206	4.03 ± 0.77	4.01 (3.52~4.4.54)
WBC(10^9^/L)	206	6.32 ± 3.01	5.69 (4.42~7.52)
HGB(g/L)	206	128.48 ± 21.33	128 (116~144)
AST (U/L)	206	363.18 ± 401.28	191 (93.50~530.25)
ALT (U/L)	206	558.43 ± 730.19	249 (87.50~675.75)
CHOI(mmol/L)	206	2.44 ± 0.88	2.33 (1.81~2.86)
ALB(g/L)	206	32.55 ± 4.99	32.20 (29.48~36.13)
CRE(umol/L)	206	65.51 ± 34.56	60 (49.75~69)
K(mmol/L)	206	3.84 ± 0.57	3.78 (3.50~4.23)
FIB (g/L)	206	1.53 ± 0.57	1.51 (1.12~1.82)
PTA(%)	206	38.47 ± 16.55	35.60 (27~46.25)
Child–Pugh class (A/B/C)	206	3/66/137	
QT (ms)	206	383.71 ± 44.34	388 (364~410.5)
QTC (ms)	206	426.84 ± 37.83	425.52 (405.99~447.83)
MELD score	206	18.84 ± 6.32	18.11 (15.39~22)

The corrected QT intervals were calculated using Bazett’s formula, and patients were stratified into 2 groups according to QTc duration: the prolonged QTc group (> 440 ms) and the non-prolonged QTc group (≤ 440 ms).

Univariate analysis demonstrated that there were no statistically significant differences between the 2 groups in terms of age, sex, white blood cell count (WBC), aspartate aminotransferase (AST), alanine aminotransferase (ALT), hepatic encephalopathy (HE), albumin (ALB), prothrombin time activity (PTA), or MELD score (*P* > .05). However, significant differences (*P* < .05) were observed in HBV-DNA level, presence of ascites, red blood cell count (RBC), total cholesterol (CHOI), creatinine (CRE), potassium (K), fibrinogen (FIB), and Child–Pugh classification, as shown in Table 2.

**Table 2:** Comparative data of HBV-ACLF patients.

Variables	QTc prolongation			No QTc prolongation			*P* value
	Number of patients(n)	Mean ± SD or Frequency (percentage)	Median (Range)	Number of patients (n)	Mean ± SD or Frequency (percentage)	Median (Range)	
Sex (male/female)	68	59/9		138	118/20		.807
HBV-DNA(>20IU/mL/<20 IU/mL)	68	58/10		138	131/7		.018
Ascites(positive/negative)	68	45/23		138	65/73		.01
HE (positive/negative)	68	10/58		138	25/113		.54
Age (years)	68	41.84 ± 10.31	41.50 (34.50~50)	138	42.95 ± 11.45	42.50 (33~51)	.703
RBC (10^12^/L)	68	3.86 ± 0.80	3.97 (3.14~4.42)	138	4.11 ± 0.75	4.03 (3.67~4.65)	.024
WBC (10^9^/L)	68	6.38 ± 3.07	5.76 (4.33~7.90)	138	6.29 ± 2.99	5.64 (4.42~7.48)	.708
AST (U/L)	68	304.30 ± 401.96	178.50 (75.75~367.25)	138	392.20 ± 399.21	212.50 (100.25~576.25)	.079
ALT (U/L)	68	438.05 ± 649.01	185 (58~517.75)	138	617.74 ± 762.29	276 (91.75~844.75)	.051
CHOI (mmol/L)	68	2.18 ± 0.84	2.03 (1.64~2.51)	138	2.57 ± 0.87	2.53 (2~3.02)	<.001
ALB(g/L)	68	31.59 ± 4.66	31.60 (28.58~34.85)	138	33.03 ± 5.09	32.80 (29.68~37.45)	.051
CRE (umol/L)	68	58.97 ± 23.70	58 (47.25~65)	138	68.73 ± 38.48	61 (50.75~74.25)	.042
K(mmol/L)	68	3.63 ± 0.58	3.65 (3.30~3.94)	138	3.94 ± 0.84	3.86 (3.57~4.28)	<.001
PTA (%)	68	35.74 ± 13.24	34.05 (26.93~40.57)	138	39.82 ± 17.85	37.85 (27~50.25)	.149
FIB(g/L)	68	1.37 ± 0.45	1.36 (0.98~1.73)	138	1.61 ± 0.61	1.57 (1.17~1.93)	.006
Child–Pugh class (A/B/C)	68	0/12/56		138	3/54/81		.001
MELD score	68	19.60 ± 4.49	18.95 (16.79~21.58)	138	18.47 ± 7.04	17.47 (14.68~22.09)	.058

Variables identified as significant in univariate analysis were subsequently entered into multivariate binary logistic regression. The results indicated that serum potassium (K), creatinine (CRE), HBV-DNA level, and the presence of ascites were independent factors associated with QTc prolongation (*P* < .05), achieving a predictive accuracy of 71.4%, as shown in Table 3.

**Table 3:** Multifactorial Analysis of QTc Interval Prolongation in Patients with HBV-ACLF.

Variables	β	*P*	OR	95%CI
K	−1.354	<.001	0.258	0.130–0.513
CRE	−0.017	.019	0.983	0.970–0.997
HBV-DNA	−1.310	.041	0.270	0.077–0.946
Ascites	0.870	.043	2.388	1.028–5.543

The forest plot is presented in Figure 2.It was generated to illustrate the independent influencing factors of QTc prolongation. The analysis revealed that ascites was an independent risk factor for QTc prolongation, whereas potassium, creatinine, and HBV-DNA acted as independent protective factors.

**Figure 2.**
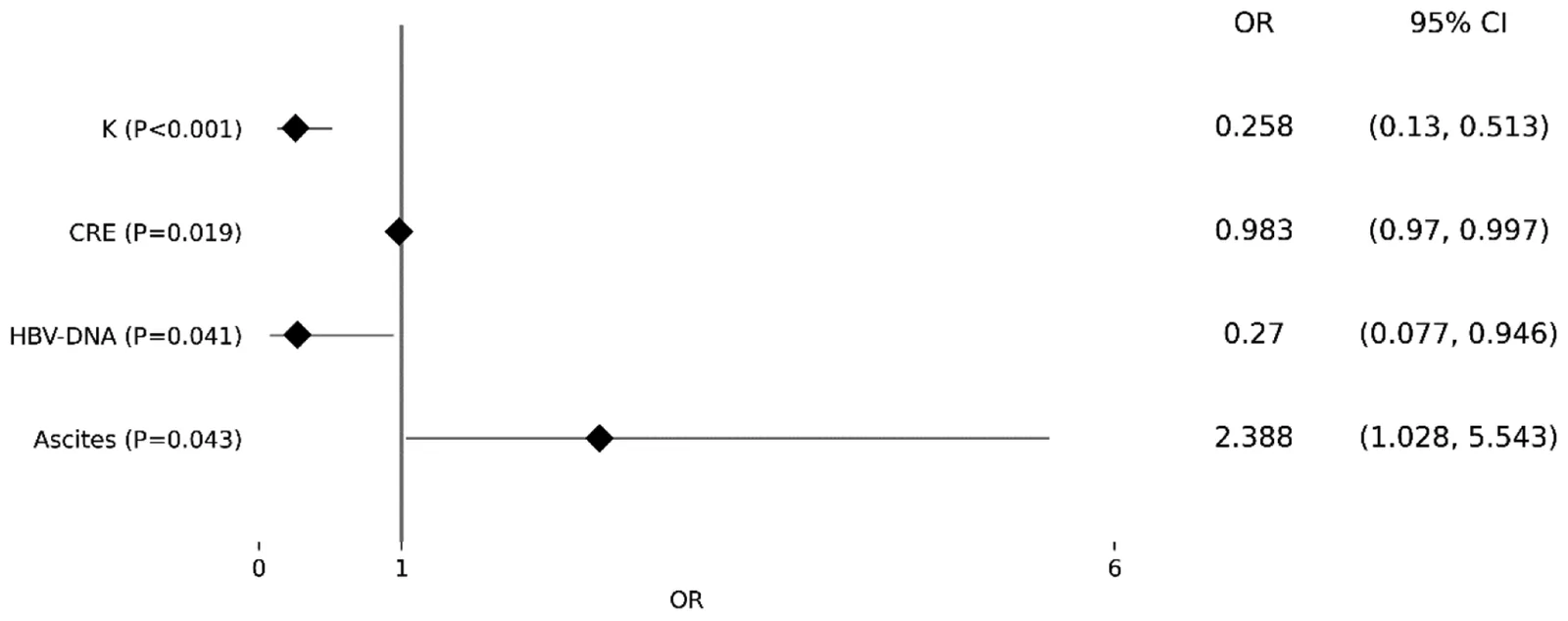
Forest plot showing independent predictors of QTc prolongation.

## 4. Discussion

To the best of our knowledge, this study is the first in China to investigate the prevalence and independent influencing factors associated with prolonged QTc intervals in patients with HBV-related acute-on-chronic liver failure (HBV-ACLF). Danish researchers previously reported that approximately 37% of cirrhotic patients exhibited prolonged QTc intervals, compared with 5.9% in healthy controls.^[12]^Similarly, a large Chinese study involving 1268 cirrhotic patients found a QTc prolongation rate of 38.2%.^[11]^Our findings revealed a comparable incidence of 33% among HBV-ACLF patients.

Age and sex are known physiological factors influencing QTc duration, with older individuals and females generally exhibiting longer QTc intervals. However, most studies have suggested that liver disease attenuates gender-related differences in QTc prolongation.^[13,14]^Our results are consistent with these observations, showing no significant influence of age or sex on QTc prolongation in HBV-ACLF patients.

Previous studies have also proposed that QTc prolongation correlates with disease severity, as reflected by Child–Pugh or MELD scores. Nevertheless, some reports failed to identify such associations, aligning with our findings.^[15,16]^In our cohort, serum potassium (K), creatinine (CRE), HBV-DNA level, and ascites were identified as independent factors influencing QTc prolongation.^[15,17]^

Recent evidence suggests that total cholesterol may affect QTc duration, particularly in specific populations such as pregnant women.^[18]^Although total cholesterol was statistically significant in univariate analysis, it did not remain an independent factor in multivariate regression, suggesting an indirect effect.

Bile acids are synthesized in hepatocytes as major products of cholesterol metabolism. In clinical settings, liver failure is frequently accompanied by elevated bile acid levels, particularly in acute or chronic forms. Experimental studies have shown that bile acids can activate nuclear receptors such as the farnesoid X receptor (FXR) and membrane receptor Takeda G protein–coupled receptor 5 (TGR5), leading to cytotoxic effects, myocardial apoptosis, and hemodynamic alterations that contribute to QTc prolongation and cardiac dysfunction.

Total cholesterol may therefore influence QTc duration indirectly through bile acid metabolism. Bile acids, which reflect the severity of liver dysfunction, have been associated with adverse outcomes in liver disease.^[19]^In our study, bile acids were not independently correlated with QTc prolongation, possibly because serum bile acids include both hydrophobic (cytotoxic) and hydrophilic (protective) subtypes.^[20,21]^Hydrophilic bile acids, such as ursodeoxycholic acid (UDCA), can counteract the cardiotoxic effects of hydrophobic species. For example, UDCA administration in cholestatic mouse models has been shown to reverse myocardial injury.^[22]^

Further investigation of specific bile acid subtypes may elucidate the mechanisms linking hepatic dysfunction to cardiac electrophysiological abnormalities. Although such mechanisms have been explored in animal studies, human data remain scarce, providing an important direction for future research.

Potassium channels play a key role in myocardial action potential generation. Systemic inflammation, which is common in liver failure, may modulate potassium channel expression through cytokine-mediated pathways, thereby affecting repolarization and QTc duration.^[23]^Multiple studies have reported lower serum potassium levels in patients with QTc prolongation compared to those without.^[24]^Variations in serum electrolyte concentration can influence cardiac excitability and conduction velocity. According to Sherif et al., each 1 mmol/L increase in serum potassium shortens QTc by approximately 16 ms.^[25]^

In clinical practice, patients with HBV-related liver failure typically exhibit reduced total cholesterol, elevated bile acids, impaired renal function, and electrolyte disturbances—all of which correlate with disease severity.^[12]^Our results demonstrated a significant negative correlation between QTc interval and serum creatinine levels (*P* < .019), consistent with prior studies.^[26]^

Given that all patients in our study had HBV-related ACLF, we also evaluated the relationship between HBV-DNA load and QTc prolongation. Using a threshold of 20 IU/mL, statistical analysis showed a significant association between viral load and QTc duration, representing the first reported link between HBV replication and cardiac electrophysiology.

Finally, our results revealed that ascites serves as an independent risk factor for QTc prolongation in HBV-ACLF patients, which aligns with findings from international studies (*P* = .043; Table 3).^[11,27]^

Collectively, these findings suggest that hepatic, renal, and electrolyte imbalances, together with systemic inflammation, may contribute to cardiac electrophysiological alterations in HBV-ACLF. Understanding these interactions is crucial for improving risk assessment, prevention, and management of cardiac complications in this high-risk population.

## 5. Conclusions

In conclusion, this study is the first to investigate the prevalence and determinants of prolonged QTc intervals in patients with HBV-related acute-on-chronic liver failure (HBV-ACLF). We found that QTc prolongation is a common electrophysiological abnormality in this population, with an incidence of approximately 33%. Serum potassium (K), creatinine (CRE), HBV-DNA level, and ascites were identified as independent influencing factors associated with QTc interval prolongation.

These findings highlight the importance of closely monitoring QTc intervals in patients with HBV-ACLF. Clinicians should pay particular attention to electrolyte imbalances, renal dysfunction, and ascites management. Early correction of hypokalemia, timely treatment of ascites, and protection of renal function may help reduce the risk of malignant arrhythmias and improve patient outcomes.

Because QTc measurement is simple, noninvasive, and repeatable, it may serve as a valuable clinical indicator for assessing arrhythmic risk and cardiac dysfunction in HBV-ACLF patients. Increased awareness of QTc prolongation and avoidance of QT-prolonging medications may further help lower mortality in this vulnerable population.

### 5.1. Limitations

This study has several limitations that should be acknowledged. First, the conclusions may have been influenced by the relatively limited sample size and the lack of statistical significance for certain clinical variables. In addition, the absence of baseline or pre-disease ECG data represents an inherent limitation, as it prevents the complete exclusion of preexisting QTc abnormalities. In clinical practice, most patients with acute-on-chronic liver failure are admitted in critical condition, and reliable pre-disease ECG records are rarely available. To minimize potential bias, we excluded patients with known cardiac disease or those taking QT-prolonging medications, ensured that all ECGs were obtained under standardized conditions using the same equipment, and confirmed that no significant intergroup differences existed in relevant comorbidities.

Second, although QTc interval analysis provides valuable information on cardiac electrophysiology, a more comprehensive cardiac assessment – including echocardiographic evaluation – would offer deeper insights into structural and functional changes. However, echocardiographic data were not available for all patients, which limited the scope of our cardiac analysis.

Finally, because of the retrospective and cross-sectional design of this study, causal relationships could not be definitively established. Future prospective studies with larger sample sizes and long-term follow-up are needed to further confirm our findings and clarify the dynamic relationship between hepatic failure progression and cardiac electrophysiological alterations.

## Acknowledgments

We thank all patients who participated in the study and the National Natural Science Foundation for their support.

## Author contributions

**Data curation:** Rou Zhang, Mengjun Fu.

**Formal analysis:** Rou Zhang, Heng Gao, Mengjun Fu.

**Writing – original draft:** Rou Zhang.

**Project administration:** Heng Gao.

**Investigation:** Mengjun Fu, Juan Li, MiMi Zhou, YaLi Feng.

**Supervision:** Juan Li, MiMi Zhou.

**Methodology:** MiMi Zhou, Yingli He.

**Visualization:** YaLi Feng, JinFeng Liu, YingRen Zhao, Yingli He.

**Writing – review & editing:** JinFeng Liu, YingRen Zhao, Yingli He.

**Conceptualization:** Yingli He.
